# Conduction in cardiac tissue. Historical reflections

**DOI:** 10.14814/phy2.13860

**Published:** 2019-01-03

**Authors:** Edward Carmeliet

**Affiliations:** ^1^ Katholieke Universiteit Leuven Leuven Belgium

**Keywords:** conduction, ephapse, gap junction, ionic theory, patch

## Abstract

Two hypotheses have been proposed to explain propagation of the action potential in heart. According to the gap junction hypothesis local short‐circuit currents pass from the proximal depolarized cell to the distal inactive cell via gap junctions and are responsible for the depolarization of the distal cell. In the ephapse hypothesis the depolarization of the proximal cell generates an electrical field in the narrow cleft between cells resulting in depolarization beyond threshold of the distal cell. Measurements of length constant, free diffusion of ^42^K, local currents between cells, existence of high‐conductance gap junctions led to the conclusion that heart muscle is a functional syncytium. Propagation of the action potential, however, is not uniform but anisotropic and discontinuous; it can be also unidirectional. These findings are strong arguments in favor of the gap junction thesis. They do not exclude, as predicted by theoretical calculations, that in conditions of an abnormal fall in gap junction conductance ephaptic conduction takes over. In this last case, definitive experimental confirmation is still required.

**See also:** https://doi.org/10.14814/phy2.13861 & https://doi.org/10.14814/phy2.13862

## Heart Muscle: Separate Cells or Syncytium?

### Conduction of the action potential is electrical

**Figure 1:**
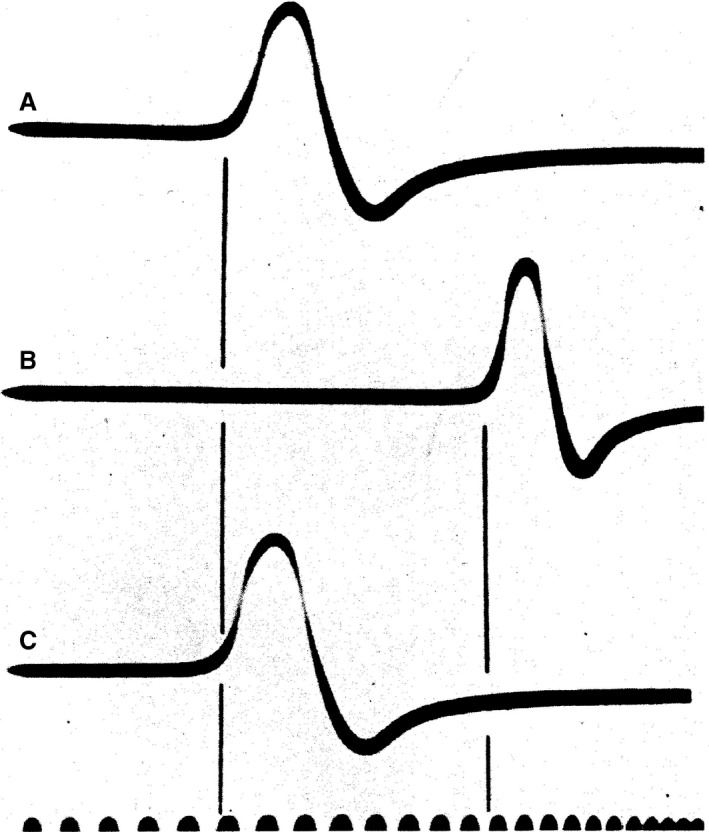
Test of the hypothesis of local circuits and conduction in the squid giant axon. (A and C) Squid giant axon in sea water. (B) Preparation in moist air. The delay between stimulus and action potential is prolonged. Conduction velocity is decreased when preparation is in a medium of higher electrical resistance. Time axis: 5000 cycles (Hodgkin 1939). With permission.

In nerve (frog n. ischiadicus) propagation, the velocity of the electrical impulse was measured by H. von Helmholtz in 1849 to be 24.6–38.4 m/sec and this value was confirmed by Bernstein (1868) using his newly developed differential rheotome. It may seem strange but at that time there was no agreement among scientists about the underlying mechanism of conduction. In his membrane theory, Bernstein (1902) proposed the mechanism of local circuit currents, a hypothesis initiated and strongly supported by Hermann (1899). In the 1930s a substantial number of biophysicists, however, were still thinking in terms of chemical transmission, with the phenomena in the cell membrane known as epiphenomena. See for example a publication by AV Hill “Chemical wave transmission in nerve” (Hill 1932). The controversy persisted until 1939 when Alan Hodgkin provided a convincing argument in favor of the existence of local currents (Hodgkin 1939). At that time Alan Hodgkin was staying in New York with Harry Grundfest at the Rockefeller Institute on a research fellowship. The test was performed in Woods Hole, Marine Biological Laboratory. The decision to perform the experiment came after Hodgin's visit with Joseph Erlanger in St Louis. Erlanger had expressed his skepticism on the local current theory for propagation. During their conversation it became clear, however, that an experimental test was possible. After his return to New York Hodgkin (1939) performed the simple, but beautiful experiments in which he measured conduction velocity in crab nerves and squid axons under conditions of different resistances of the extracellular solution (oil, moist air, Ringer solution). Conduction velocity was fastest when the extracellular resistance was lowest (Fig. 1). Erlanger was convinced that local currents were the right answer for the mechanism of action potential propagation. Values for conduction velocity in nerve varied between 1 and 100 m/sec and in heart a 100 times slower. The subsequent elucidation of the ionic theory in 1952 was the definitive end of the chemical theory of action potential propagation. It may be worthwhile to stress that Hodgkin reduced the volume of fluid around the nerve to increase the extracellular electrical resistance; his method was the easiest way to modify resistance without changing the ionic composition and thus avoiding in this manner any modification of excitability. Changes in excitability should be considered when the effect of experimentally induced edema on conduction velocity is studied.

### Histological measurements

From histological studies with the light microscope, back in the mid of the 19th century by Kölliker (1859) and Eberth (1866), cited by Forbes and Sperelakis (1985) it was known that thick transverse bands, heavily stained, situated at the longitudinal ends of two cells, interrupted the striated pattern of the muscle at regular intervals. The early workers correctly identified the disks as points of apposition of individual cell boundaries. It is the only site where cells touch each other. They were called intercalated disks but their function was not clear. Were these the functional connections between cells? On this aspect there existed controversy in the late 19th century with two opposing theses: according to one group the heart was a syncytium with free access from cell to cell. According to the other group it consisted of individual cells, isolated from each other by high‐resistance membranes. A first step in the direction of functional syncytium was made by measuring the length constant.

### Measurement of length constant

From 1939 on, propagation of the axon action potential was considered a physical process originating in the membrane. Quantitative considerations were based on the cable theory developed by William Thomson (Lord Kelvin, 1824–1907), on the occasion of the laying of an underwater sea cable in the Atlantic Ocean. An important basic constant in cable theory is the length or space constant *λ*. Quantitatively it depends on rm, the membrane resistance and ra the intracellular resistance according to the equation

$$\lambda =\sqrt{\text{rm/ra}}$$

It describes the exponential spread of a charge injected into the cable. At distance *λ* and in steady state the potential will drop to 1/e, which is about 0.37 times the potential at the site of current injection. The phenomenon is called electrotonus and is considered the basic mechanism of propagation in excitable tissues. The greater *λ*, the further away from origin the current will travel in a given time, eventually to reach threshold and determine conduction velocity.

Application of the cable theory to axons may appear self‐evident because of the cable‐like structure of axons. But what about cardiac muscle? The first measurements of *λ* in cardiac Purkinje fibers were made by Weidmann (1952) and found to be 1.9 mm. This value is much longer than a cell length and thus suggests that the injected current has spread over more than one cell. In other words current can pass through cells as if there are cytoplasmic bridges between them. Or in other words the cardiac muscle is a functional syncytium. Later measurements in other cardiac tissues confirmed the observation that *λ* was longer than the cell length (0.88 mm in trabecular muscle of sheep and calf (Weidmann 1970), 0.215 mm in rat auricle (Hervé et al. 1989), and 0.357 mm in rabbit papillary muscle (Kléber and Riegger 1987).

Silvio Weidmann was one the firsts to try to measure the electrical resistance of the intercalated disk by using two double‐barreled electrodes (Weidmann 1964). As he mentioned in the reports of a symposium in October 1963, the experiment was entirely unsuccessful. Failure, however, is often the start of success. It was indeed this failure which forced Silvio to begin the experiments with ^42^K^+^, which were a great manifestation of technical craftsmanship and performance and provided basic information on the process of action potential conduction in the heart.

### K^+^ ions diffuse freely between cells

**Figure 2:**
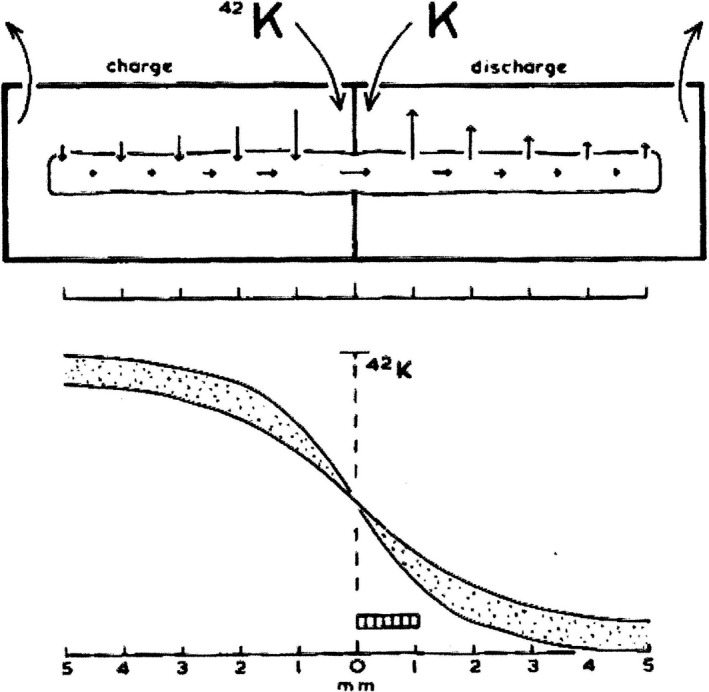
Do K^+^ ions diffuse freely from cell to cell? One half of a myocardial strip (trabecula carnea of sheep ventricle) is loaded with ^42^K^+^, the other half washed by inactive solution. The equilibrium distribution of radioactive K^+^ over the length of a cardiac muscle preparation follows the rules of the cable equation. (Weidmann 1964, 1966). With permission.

The spread of electrotonic current suggests that flow of ions from cell to cell obeys the law of a free diffusion process. The existence of an easy passage has been confirmed by measurements of the distribution of radioactive ^42^K^+^ in trabecular muscle preparations (Weidmann 1966). In these experiments one half of a myocardial strip (trabecula carnea of sheep ventricle) was loaded with ^42^K^+^, the other half washed by inactive solution. When diffusion equilibrium was reached the bundle was dropped into liquid air or isopropane. It was sliced into short bids of 0.5 mm which were counted for radioactivity (Fig. 2). In a second series of experiments Weidmann not only measured the distribution of ^42^K^+^ in steady state but was able to follow the building up during exposure of radioactive solution followed by the decline in time during washout. By applying the cable equation, length constants were calculated and found to range between 1.3 and 2.2 mm (20 experiments), similar to values obtained for electrically determined length constants. Disk resistance was estimated to be 500–1500 times lower than the resistance for unit area of the cell membrane. The results of the electrotonic as well as the ^42^K^+^ efflux experiments have been interpreted as demonstrations that heart cells communicate with each other and function as a syncytium.

### Sucrose gap and local currents

**Figure 3:**
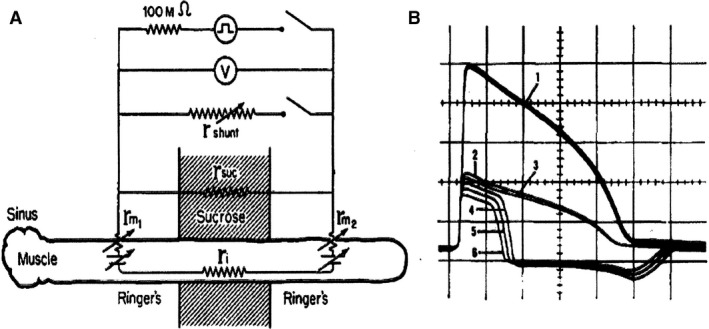
Propagation of the cardiac action potential depends on current through the gap junctions between cells. (A) Schematic representation of the experimental design. (B) Electrical records in the absence of a shunt resistance (1) and presence of decreasing shunt resistance (2–6). At shunt resistance 4: the monophasic action potential changes in diphasic, meaning that threshold is reached in the right part of the preparation. Confirmation was made by the presence of a mechanical contraction (not shown). Divisions: 20 mV and 100 msec. Gap width: 400 *μ* (Barr et al. 1965). With permission.

The existence of a free pathway for diffusion of ions as shown by the ^42^K^+^ flux experiments suggests propagation of the action potential via local current circuits in the extracellular medium. The existence of local currents in nerve fibers was already demonstrated by Hodgkin in 1939. For heart muscle an elegant demonstration was provided by Barr et al. (1965). These authors used a frog atrial bundle or a guinea pig papillary muscle mounted in a sucrose gap setup as shown in Figure 3A. When the preparation is stimulated a monophasic action potential (Fig. 3B) is recorded between the active electrode and the indifferent electrode. Propagation from left to right is blocked by the high extracellular resistance of the sucrose solution, which prevents local currents to reach the right part. After connecting both parts of the muscle by a shunt resistance, a circuit between both parts of the preparation is closed via the intracellular pathway in the sucrose gap. The resistance of the shunt, however, is still too high to allow sufficient current to reach the right part. By gradually decreasing the shunt resistance more current can reach the right part of the preparation. The electrical signal upon stimulation of the left part first becomes smaller in amplitude and then changes into a biphasic recording (Fig. 3B). The biphasic nature of the electrogram demonstrates that threshold has been reached and the right part has been excited. The presence of activation was proven by recording the development of mechanical tension (not shown in the figure). This was a further indication that current could easily pass from one cell to another, even if the extracellular pathway is blocked.

**Figure 4:**
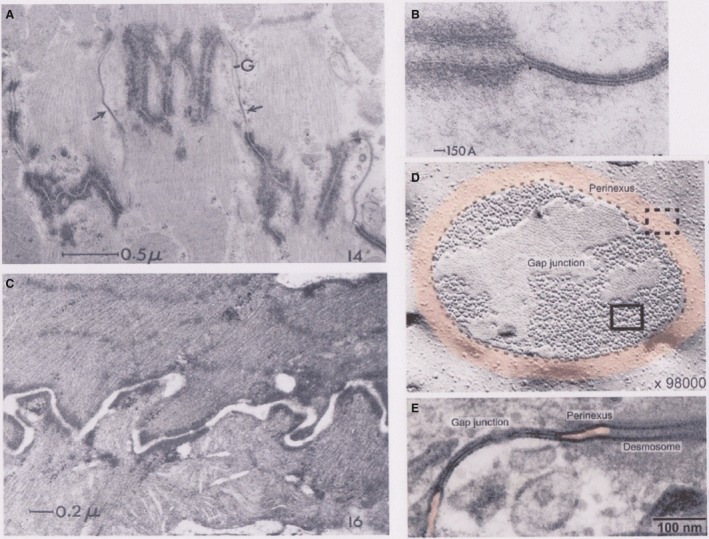
Intercalated disk guinea pig papillary muscle. (A and B) Visualization at two different magnifications. Myofibrils attach to the membranes of adjacent cells at the interfibrillar region of the disk as finely fibrous masses, normal to the longitudinal axis of the cell. The space between cell membranes is from 100 to 200 Å. In the intersarcoplasmic regions of the disk, which here lie parallel to the fiber axis, nexuses (arrows), and desmosomes occur along the adjacent membranes. In addition, a gap between the membranes greater than 100 Å is always present in the nonnexal regions of this portion of the disk (Barr et al. 1965). (C) Effect of incubation in hypertonic sucrose solution on the intercalated disk. The gap between adjacent cell membranes along the disk widens with complete rupture of the nexuses. This phenomenon is reversible (Barr et al. 1965). (D) The perinexus. Freeze fracture electron micrograph showing en face view of a gap junction plaque in ventricular myocardium (outlined by dashed black line) surrounded by perinexus (tinted orange). Individual connexons, seen as particles are densely packed in a hexagonal array within the gap junction (solid rectangle) but are less concentrated and organized within the perinexus (dashed rectangle). (E) Transmission electron micrograph of gap junction in ventricular myocardium flanked on either side by perinexus (orange tinted). A desmosome is also visible on the right hand side (Rhett and Gourdie 2012). With permission.

Another demonstration of special importance by Barr et al. (1965) is that the hypertonic sucrose solution blocked the passage of current through the gap between both parts of the preparation, in a reversible way. In control electron microscopic photomicrographs of preparations subjected to hypertonic sucrose, it was shown that the nexus was ruptured in a reversible way. Compare normal intercalated disk in Figure 4A with disrupted disk in Figure 4C. This strongly suggests that the nexus is responsible for the low resistance between cardiac cells. The two parts of the gap junction can be separated by hyperosmotic solutions but recovery is rapid. The nexus is that part of the intercalated disk where free access from cell to cell is guaranteed.

### Description of the gap junction

The first descriptions of the nexus and gap junction channels with the electron microscope, however, were rather supporting the other thesis, that is, heart is composed of individual cells isolated from each other. A century after the light microscopic analysis, Sjöstrand and Andersson (1954), using thin slices of cardiac preparations presented an electron‐microscopic picture of the cardiac muscle composed of individual cells surrounded by a complete and continuous membrane. In their analysis the authors specifically emphasized that the cells were isolated from each other. Cytoplasmic continuity between cardiac cells did not exist. Conductance as a pathway was transformed to a resistance.

Further electron microscopic studies provided a picture of the intercalated disk consisting of three elements (Barr 1965; van der Velden and Jongsma 2002): fascia adherens, desmosome, and gap junction. The fascia adherens and desmosome are mechanical junctions which protect the cells against excessive stress; fascia adherens is furthermore an insertion site for myofilaments. The third or gap junction, first described by Revel and Karnovsky 1967, consists of a variable number (a few to more than thousands) of gap channels in a plaque (Fig. 4D). Each channel consists of two connexons (hemichannels); and each connexon of six connexins. (Brightman and Reese 1969; Goodenough 1974) coming from two different cells. In cardiac cells there exists three main connexins: CX40, CX43, and CX45. The very close apposition of the membranes of two cells (Fig. 4A, B, and E) was due to the presence of channels that were responsible for mechanically joining the two cells but at the same time providing a free conductive pathway. Many scientists work late. The discovery of the gap junction in cardiac cells was made at 2.00 am by Karnovsky who got his first tangential sections with the hexagonal packing of gap junctions in cardiomyocytes. Karnovsky took the wet plates to show them to Revel his coauthor in the neighboring lab. Later similar contact zones were found in smooth muscle (Dewey and Barr 1962) and many other cells. Electrophysiologic investigations of these plaques revealed the existence of channels characterized by a high conductance. The permeability of connexin channels, however, was not restricted to passage of ions but the channels also serve as a pathway for metabolites and messenger substances like cAMP, IP3, and substances with a molecular weight up to 1.2 kD.

## Heart Muscle is a Functional Syncytium but Conduction is not Continuous

Emphasizing cable behavior may have given the wrong impression that conduction of the action potential occurs in a simple continuous medium. This is not the case. The impulse in the course of its spread over the whole heart shows conduction velocities which are low in the sino‐atrial and auriculo‐ventricular node, faster in atrium and ventricle, and fastest in the His–Purkinje system. The differences are due to morphological and functional properties (e.g. diameter of cell, number of cells, expression of channels, ionic currents).

### Anisotropic conduction

An important characteristic feature especially with regard to the genesis of arrhythmias is the difference in conduction velocity between the longitudinal and transverse direction in a bundle of parallel muscle fibers. Conduction was found to be much faster in the longitudinal compared to the transverse direction. The conduction velocity is said to be anisotropic. This finding was not entirely unexpected, because the structure of the cell is anisotropic: length and width are different in dimension and the relative amount of cytoplasm and membrane is thus different per unit length. The first publications on this characteristic feature were made by Sano et al. (1959) who measured in dog epicardial muscle a conduction velocity of 7–70 m/sec in the longitudinal direction and 8–20 m/sec in the transverse direction. Draper and Mya‐Tu (1959) used goat, dog, cat, and rabbit. Their description was rather limited, but the authors noted that values in the longitudinal direction varied between 0.4 and 0.9 m/sec; in the transverse direction velocity was less regular and smaller.

Allen Scher and collaborators (Roberts et al. 1979) recorded a conduction velocity of 58 cm/sec in the longitudinal direction and of 25 cm/sec in the transverse direction of dog ventricular tissue. Gross tissue resistivity was 3.2 times smaller in the longitudinal direction, and the voltage across the depolarization wave in the transverse direction was approximately three times as great as in the longitudinal direction.

An analysis by Clerc (1976)) in the right ventricle of the calf shows a ratio of 3/1 for longitudinal to transverse conduction velocity, with absolute values of 48 and 16 cm/sec. According to Clerc the disparity in conduction velocity could be explained on the basis of differences in resistivity. The ratio for intracellular resistances transversal to longitudinal was 9.4, and for extracellular resistances 2.7. Compare with results from Scher et al. above.

Propagation of the action potential was conditioned by the passive spread of electric charges along the cable. Clerc especially emphasized that the action potential should be considered a source of current spreading to the adjacent tissue. The adjacent tissue then acts as a sink. The amount of charge in the source (under normal conditions carried by the sodium current) should be large enough to bring the sink to threshold. The ratio of these two charges is called the safety factor and should be 1.0 or greater.

### Conduction is discontinuous

High velocity of conduction has usually been thought to be associated with a high safety factor (Spach et al. 1981; Spach 1999). Spach (1999), however, discovered that this combination is not always valid. According to his analysis propagation at low velocity is more resistant to disturbances in membrane properties than propagation at high velocity. Conduction is thus not uniform but discontinuous. At the microscopic level, conduction is better described as saltatory, whereas at the macroscopic level it seems continuous. The situation has thus changed to: high conduction velocity in the longitudinal direction combined with low *V*_max_, and low safety factor. In the transverse direction: conduction velocity is low, *V*_max_ and safety factor high. This peculiar relationship suggests that when longitudinal propagation is blocked because of low safety factor the impulse can eventually continue in the transverse direction and be the start of reentry (Spach 1999).

**Figure 5:**
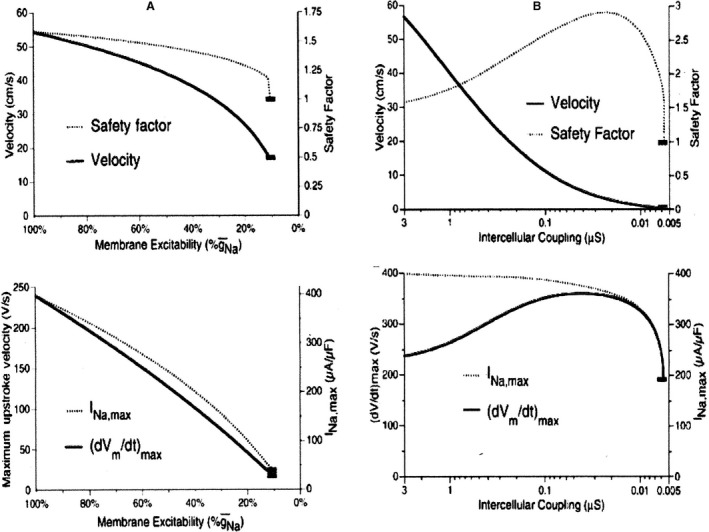
Theoretical calculation of the effect of reduced membrane excitability (A) and of gap conductance (B) on four conduction parameters: safety factor, velocity of conduction, INa^+^ max, and *V*_max_ of action potential upstroke. Note logarithmic scale for gap conductance (Shaw and Rudy 1997). With permission.

The “inverted” relationship between high conduction velocity and low safety factor has been the topic of a theoretical study (Shaw and Rudy 1997) see Figure 5. According to this analysis propagation should be regarded as a complex phenomenon that involves the interaction between membrane currents, gap junction properties and intracellular ionic processes. The effect of a decrease in membrane excitability (Fig. 5A) and of intercellular coupling (Fig. 5B) on four parameters of propagation was studied: conduction velocity, safety factor, *V*_max_, and INa^+^ max. With a fall in excitability, which for instance can be seen in the presence of the class 1 antiarrhythmics, Na^+^ conductance and *V*_max_ gradually decrease resulting in a reduction of safety factor and conduction velocity. Here safety factor and conduction velocity go hand in hand. Velocity which at the start was 54 cm/sec stops at 17 cm/sec, that is, at one‐third of its normal value. The Na^+^ current intensity is then about 11% of normal. The predominant current up to 20% of the maximum Na^+^ current is still the Na^+^ current, and Ca^2+^ current participation remains low.

**Figure 6:**
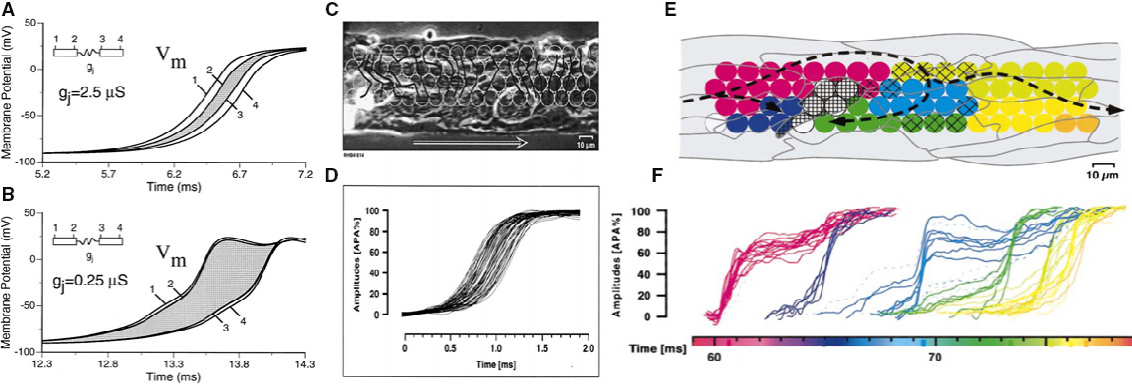
(A and B) Calculated effect of a decrease in gap junction conductance on conduction from cell to cell. (A) Normal gap junction conductance is 2.5 *μ*S. (B) Gj is reduced to 0.25 *μ*S. For normal coupling (A) intercellular conduction delay at the gap junction is approximately equal to the intracellular conduction time. A tenfold decrease in gap conductance (B) increases the intercellular delay and decreases the intracellular conduction time drastically, resulting in gap junction dominance of macroscopic conduction velocity. (Shaw and Rudy 1997). (C) and (D) Experimental measurement of microscopic conduction under control conditions. (C) Phase contrast image of the preparation with superimposition of the white circles indicating the location of the photodetectors. Stimulation on the left side. Conduction velocity 43 cm/sec. (D) Superimposition of all action potentials measured by the photodetectors (Rohr et al. 1998). (E) and (F): Action potential propagation during marked cell‐to‐cell uncoupling in a synthetic strand of neonatal rat myocytes. (E) image of a segment of the strand with colored circles denoting position of light‐measuring diodes. Path followed by the excitation (F) action potential recorded by each diode, shows clustering of upstrokes in groups. Within each group excitation is practically simultaneous; propagation delays up to 5 msec occur between groups. Note that a number of cells are excited almost simultaneously and that complete conduction block occurs in the cell marked by the rectangular hatching. Delays are confined to cell borders (Rohr et al. 1998). With permission.

With a fall in intercellular coupling, as that which occurs in the presence of heptanol or palmitoleic acid, Na^+^ current remains practically constant over a broad range of coupling values (see logarithmic scale). A calculated example of how the action potential travels from cell to cell is shown in Figure 6A and B. Action potential upstrokes from the edge elements of neighboring cells are shown. Intercellular coupling is assumed to fall from 2.5 to 0.25 *μ*S. With normal coupling the action potential spends as much time in the gap as in the intracellular space. In reduced gap conductance the time lost in the gap is much increased and propagation is delayed, while propagation in the cell cytoplasm is enhanced. Conduction becomes saltatory.

Rohr et al. 1998 (Rohr et al. 1998) measured conduction velocity in linear cell strands (55 *μ*m width) under normal conditions and after treatment with palmitoleic acid, a gap blocker (Fig. 6C–F). In normal Tyrode conduction velocity was 43 cm/sec and uniform as indicated by the mostly parallel and evenly spaced isochrones and smoothly rising upstrokes, showing no major interference with the cell borders. After treatment with the gap blocker the situation is totally different (Fig. 6E and F). Conduction velocity dramatically decreases to 1.1 cm/sec, activation advances stepwise along the preparation as indicated by the clustering of the upstrokes. Intercluster delays are going up to a few msec and illustrate the presence of highly discontinuous conduction. The spatial origin of the clustered signals is illustrated in Figure 6E. Clustered activity originated from small patches consisting of 1–3 cells. Activation was tortuous, and even sometimes backwards because of cells which did not activate. Meandering of the impulse was thus present at a microscopic level. Conduction was now dependent on Ca^2+^ action potentials. The occurrence of Ca^2+^‐dependent activity (Rohr and Kucera 1997) was also demonstrated in other experiments where conduction velocity was drastically reduced by propagation of the impulse from a narrow linear strand into an abrupt tissue expansion. Anterograde block could be relieved by local perfusion with a solution containing BayK8644, suggesting the intervention of Ca^2+^‐dependent electrical activity.

Under normal conditions, *V*_max_ and safety factor change in a biphasic way as a function of falling coupling conductance: both first increase to a maximum before their final fall; at their maximum, the coupling conductance decreased to about one‐hundredth of the initial value (Fig. 5B). Inward Na^+^ current in those circumstances is used for two purposes: local depolarization and axial current downstream. The latter component is reduced by the fall in g_j_; the part saved is used to upgrade *V*_max_ and safety factor upstream. Due to the increased gap resistance the axial Na^+^ current downstream is slow and accompanied by a degree of inactivation. Before finally resulting in block of conduction, an important part of the current is guaranteed by the Ca^2+^ current during the plateau of the action potential. Conduction velocity falls gradually over the whole range of coupling resistances and attains very low values of 2 cm/sec before block. With a fall in excitability on the other hand block occurs at much higher conduction velocity of about 20 cm/sec.

### Unidirectional conduction, vulnerable period, reciprocating waves, and fibrillation

Electrical coupling by gap junctions is bidirectional, and propagation of the action potential can therefore proceed in two directions. Unidirectional conduction can originate from certain structural or functional characteristics, such as: (1) source–sink impedance mismatch caused for example, by a sudden increase in geometrical dimension, (2) dispersion of action potential duration, as seen in the junction of muscle and Purkinje cells, and (3) stimulation during the vulnerable period (Mines 1913a).

The analysis of the vulnerable period and its role in the genesis of fibrillation is one of Mines’ major discoveries. He described it in the ray, frog, and rabbit heart by using man‐made rings of auricular and ventricular tissue. Applying single stimuli at variable intervals during the relative refractory period results normally in an extrasystole propagated in two directions. There exists, however, a short period during which the propagation of the extrasystole is blocked in the anterograde direction, but is occurring only in the retrograde direction. Conduction becomes thus unidirectional. The reason for this difference was given by Mines in terms of a recovery process. Presently we know it concerns the recovery of Na^+^ conductance (Shaw and Rudy, 1995).

**Figure 7:**
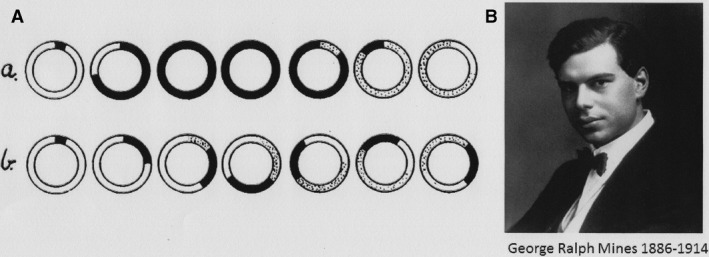
(A) Diagrammatic representation by Mines (Mines 1913a) to demonstrate circulating rhythm in closed circuits with unidirectional conduction at the start. (a) Normal tissue with rapid conduction and no reentry. (b) Abnormal tissue with slow conduction and short refractory period (dotted areas) permitting reentry to occur. Reproduced with permission. (B) George Ralph Mines (1886–1914), about 27 years old. Photograph provided by the Department of Physiology, Development and Neuroscience, Cambridge University, UK, with permission.

In case of unidirectional conduction the single extrasystolic wave can continue its way around the circle for an indefinite time. Note that extrasystolic stimulation in a circle structure is only possible when the size of the circle is large enough compared to the conduction velocity and the refractory period. High conduction velocity and long refractory period results in collision of the two waves and depolarization of the whole circle (Fig. 7A). The question can be asked whether circle structures exist in the heart. Mines was convinced: “No fact is better established concerning the histological structure of cardiac muscle than that there exist numerous connexions between the various portions or columns of cells. There exist therefore closed circuits in the myocardium.”

Already in the 19th century, it was thought that application of a single stimulus during the vulnerable period could cause tachycardia or fibrillation and be responsible for sudden death. Later experiments by Cranefield et al. (1957) demonstrated that this period coincided with the time corresponding to the dip in the excitability curve for anodal stimuli.

The phenomenon of circus movement is not restricted to the vertebrate world. In his paper of 1913 Mines refers to A. G. Mayer: “The circulating rhythm here described is precisely comparable to the state of affairs produced in rings cut from the bells of Medusae.” Experiments by Mayer were performed on Medusae bells that had been cut into rings. Application of a mechanical, chemical, or electrical stimulus resulted in pulsation that most of the time was repeated and stable for a long time. Once started it continued indefinitely in normal sea water, without further external stimulation. Activity required a closed circuit. Breaking the circuit by a cut stopped the activity immediately. The rate of pulsation depended on the length of the circuit, not on the mass or the area of the tissue. Short circuits pulsed more rapidly than did long ones. All these characteristics are typical for a circulating wave.

I find justified to digress shortly on the biography of George Ralph Mines (1886–1914) Figure 7B. He was born in 1886, entered Sydney Sussex College, Cambridge on a grant, and was elected fellow at the same college in 1909. In the same year he married Marjorie Rolfe. The couple got three children. The family was art‐oriented. M. Rolfe was a productive poet; one daughter, Anatole, became an excellent viola player and Ralph himself was gifted at the piano and had considered a career in music before deciding for physiology. In 1910 Ralph was elected together with his friend A. V. Hill as the member of the Physiological Society. Between 1910 and 1914 Mines had four productive years with over a dozen of papers published in the Journal of Physiology. He was accompanied in the same volumes by names that ring a bell: Langley, Adrian, Hill, Starling, Bayliss, Sherrington, and (Thomas) Lewis. In his scientific work he was known for making his own recording instruments and devices. He applied the latest innovations in cinematography and photography. For recording the contractions of the frog heart he was photographing at 15 frames per second on bromide paper (Mines 1913b). During experiments, he used his own Chronodictaphone to record the progress of the procedure.

Due to the shortage of qualified physiologists Mines went temporarily to Toronto (Canada). During that stay he had been invited to Montréal, at the MacGill University and had accepted the post of Professor and Chair in Physiology. In late 1914 he returned to Montréal, and although only just promoted he was asked to present the Founders’ day address on October 6. In that address, which was highly estimated, he had a section on the future of physiology and referred to the possibility of self‐experimentation. More specifically he mentioned the study of sensation by Head, who severed nerves in his arm, and a study on digestion by Washburn in Cannon's lab who swallowed a stomach tube cited by (DeSilva 1997).

On November 7, 1914, the night janitor of the institute found Ralph Mines lying unconscious with equipment attached, apparently for recording respiration. Mines died in the hospital after regaining for a short time consciousness. The autopsy did not bring a clear diagnosis. George Ralph Mines was buried on the Mont Royal Cemetery in a grave marked only by a very low headstone indicating that he died during self‐experimentation.

Alfred G. Mayer (1868–1922), cited by Mines for his work on circus movement in rings of Medusae bells and also an exceptional figure, was born in 1868 in a family of German origin. Upon the advice of his father he studied physics, but after several years of physics‐related work in a number of Universities he shifted to Biology. He studied at Harvard University, joined the laboratory of Prof L. Agassiz, a Swiss‐American biologist‐geologist, and obtained a degree in Biology (1897). He became museum curator at the Brooklyn Institute of Arts and Sciences. In 1904 he founded the Marine Biological Laboratory on the Tortugas Islands Fla., Carnegie Institution of Washington. Mayer was the first Director. The station became the most thoroughly equipped marine biological station in the tropical world. He was elected as Member of the National Academy of Sciences. He was fascinated with coral sea life and published important contributions on the subject: Rhythmical Pulsation1906; Medusae of the world, 1910; Ctenophores of the Atlantic Coast of North America, 1912; Sea Shore Life; The history of Fiji.

## Ephapse or Gap Junction Conduction?

At the present time not everyone accepts the local current hypothesis in combination with the gap junction as a satisfying explanation for the conduction process, as evident from the following statement by Nick Sperelakis at the beginning of a paper (Sperelakis 2002): “A long‐standing dogma in basic electrophysiology of the heart has been that the atrial and ventricular myocardial cells are interconnected by low resistance pathways mediated by gap‐junction connexon channels. The scientists responsible for this dogma are Weidmann, Crill and Woodbury, Carlos de Mello. This dogma has become ingrained in most textbooks and advanced reference books dealing with the heart”.

A growing group of researchers lead by Nick Sperelakis (1930–2013) (Sperelakis 2002) are convinced that conduction can be explained by the ephapse hypothesis. Some are going a step further and pretend that gap junctions are not required at all (Rhett et al. 2013).

The term “ephapse” originates from Arvanitaki 1941 and was used to describe the geometrical arrangement of very narrow extracellular spaces between unmyelinated nerves. The Greek noun means “touching, caressing”. Nerve fibers touching each other modify their excitability during activity. Experimental studies by Katz and Schmitt (1940) on Carcinus maenas nerves submerged in paraffin oil showed that a depolarization in an active nerve caused a hyperpolarization in the “touching” nerve at rest resulting in a decrease of excitability. This initial period is quickly followed by the opposite change to an increase in excitability. Simultaneous firing in two nerves provokes a fall in conduction velocity but a slight offset in the stimuli favors synchronization. More recently Ramon and Moore (1978) restudied the problem of possible ephaptic effects in paired squid giant axons. The preparations were surrounded by a thin layer of sea water and submerged in a pool of oil. Ephaptic transmission was possible when the layer of sea water around the axon‐region under investigation was made as thin as possible and kept under oil. Under those conditions extracellular potentials as high as 80 mV were recorded. Elicited transmembrane action potentials had a normal appearance. Similar results have been obtained in cylindrical preparations of the ferret heart muscle by Suenson (1984). Extracellular potential differences were larger than 60 mV. Excitability and conduction velocity were variable. Latency to threshold for instance changed between 9 and 369 msec. Various rate‐dependent blocks occurred when stimulated above 1/sec. In a study by Weingart and Maurer (1988) on cell pairs of rat and guinea‐pig ventricular cells the authors were unable to cause transmission of the action potential by gently pushing two individual cells close together.

The conclusion of these experiments is that ephaptic conduction as a mechanism is possible, but precarious. The major problem is the absence of a testable model.

A step forwards has been made recently by the proposal of a structure acting as ephapse (Rhett et al. 2013). A structure in the intercalated disk may fulfill this request. At the periphery of the gap junction a region exists where the two membrane leaflets (proximal and distal) remain close together with a narrow and tortuous space in between. Such space probably has an elevated resistance, one of the requirements for generating a high electrical field. It was given the name “perinexus” (Fig. 4D and E) and chosen as a candidate for the ephapse.

According to theoretical studies (Kucera et al. 2002) ephaptic propagation requires a substantial electric field and this should be produced by the activity of Na^+^ channels in the narrow extracellular clefts. The presence of an efficient number of Na^+^ channels is thus the second requirement for a structure to function as an ephapse. Na^+^ channels have been found to be concentrated in the perinexus (Maier et al. 2004) but are also present in lateral membranes. Cx43 and Nav1.5 participate in macromolecular complexes such as the desmosal protein plakophilin and ankyrin (Sato et al. 2011) and SAP97 at the intercalated disk (Petitprez et al. 2011), while syntrophin–dystrophin is concentrated at the lateral membranes.

**Figure 8:**
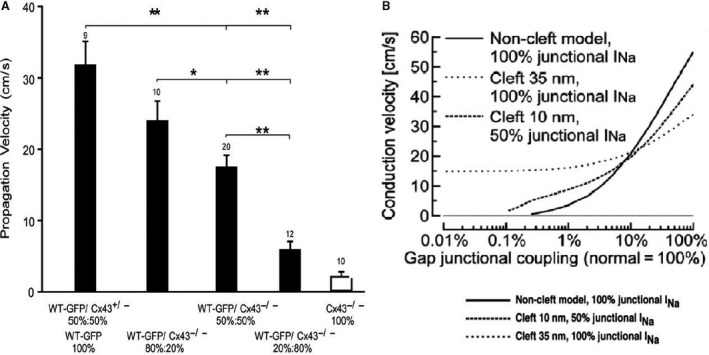
(A) Propagation velocities along strands engineered from mixed suspensions of WT (wild‐type) cells and Cx43−/− cells (knockout of the gene Cx43). Mixtures indicated on the abscissa. Propagation decreases in proportion to the contribution of Cx43−/− cells. Note the marked effect when the ratio of WT cells falls to 20% (Beauchamp et al. 2012). (B) Calculated dependence of conduction velocity on gap junction coupling for three different models of cleft configuration and partitioning of sodium channels. Solid line: model with no cleft effects but clustering of all sodium channels at intercalated disk. Dashed line: model with 10‐nm wide cleft and even partitioning of the sodium channels between the intercalated disk and the surface sarcolemma. Punctuate line: model with a 35‐nm wide cleft and clustering of all sodium channels at intercalated disk (Kucera et al. 2002). With permission.

Calculations on models with colocalization of gap junction and Na^+^ channels (Kucera et al. 2002), have shown that the presence of Na^+^ channels in intercalated disk causes a negative potential in the space between cells whose amplitude influences conduction in two opposing ways, depending on the coupling conductance (Fig. 8B). (1) with a normal or moderately reduced coupling, the negative cleft potential led to a large overshoot resulting in a decreased driving force for INa itself (self‐attenuation), which slowed conduction; (2) for greatly reduced coupling, the negative cleft potential led to suprathreshold depolarization of the postjunctional membrane, which facilitated and accelerated conduction. These computations have been repeated and confirmed by (Lin and Keener 2010) who conclude: In agreement with previous studies, we find that ephaptic coupling increases propagation velocity at low gap junctional conductivity while it decreases propagation at higher conductivities.

Very recently a study by Kucera and collaborators (Hichri et al. 2018) has added new experimental and theoretical information on the ephaptic hypothesis. The authors show that distribution of Na^+^ channels in clusters potentiates ephaptic interaction in the intercalated disk especially when clusters face each other across the intercellular cleft. In patch clamp experiments with HEK cells stably expressing Nav1.5 channels they showed that approaching a nonconducting obstacle and thus reducing the extracellular space, increased peak Na^+^current when applying a voltage step close to threshold, but reduced the current when applying a voltage step far above threshold. It was also confirmed that potentiation of the ephaptic process occurred when gap conductance was low. Hopefully more tests of this type will be possible in the future.

### Expression of Cx connexins and conduction

During the last twenty years many experiments in the field of conduction have been performed using genetic modulation of the expression of connexin molecules. The experiments with mice models and heterozygote or conditional knockout Cx43 expression provide rather restrictive and often incomplete information.

In an attempt to facilitate the gathering of data, Kléber and collaborators instead of using the mice‐animal model shifted to synthesized strands and cell pairs of ventricular myocytes from mice with altered Cx43 content. In a first series of experiments (Beauchamp et al. 2004) the Cx43 content was changed in homogeneous way and comparison was made between WT, Cx43+/−, and Cx43−/−. Cell pairs were used to measure gap junction conductance, Gj, and synthetic strands to measure conduction velocity. In CX43+/− preparations intercellular conductance was down by 32%, but propagation was not significantly changed. In the Cx43nul preparations on the other hand intercellular conductance was down by 96% and propagation was very slow: 2.1 versus 52 cm/sec in Cx43+/+, and highly discontinuous, with simultaneous excitation within and long conduction delays (2–3 msec) between individual cells. Remaining propagation was abolished by heptanol, an indication that a residual junctional coupling existed which was caused by the presence of CX45 (experimentally verified). The experiments on the heterozygote cell pair preparation confirmed the former results on the heterozygote mice model. And the very slow conduction velocity remaining in the total absence of Cx43 could be shown to be caused by Cx45. The final conclusion of the authors was: “Electric field effect does not contribute to propagation in synthetic strands”.

In a second type of experiment mixture cultures were made of germline Cx43−/− and Cx43+/+ cells. Expression was heterogeneous. Results were straightforward: conduction velocity decreased with increased proportion of Cx43 null cells (Fig. 8A). Propagation was well‐maintained down to levels of 50% Cx43 null and 50% wild‐type myocytes but drastically went down when less than 50% WT cells participated. Propagation was maintained by fast propagation meandering across C43 wild type cell clusters and discontinuous delayed propagation within areas of CX43 null cells. All cells remained excited, however, with no evidence of block. This explains why contractility in this preparation remains intact (Beauchamp et al. 2012). The general conclusion is that communication and conduction between cells occurs via gap junctions.

## Conflict of Interest

None declared.
